# Global Methylation Patterns and Their Relationship with Gene Expression and Small RNA in Rice Lines with Different Ploidy

**DOI:** 10.3389/fpls.2016.01002

**Published:** 2016-07-21

**Authors:** Hong-Yu Zhang, Hui-Xia Zhao, Shao-Hua Wu, Fang Huang, Kai-Ting Wu, Xiu-Feng Zeng, Xiao-Qiong Chen, Pei-Zhou Xu, Xian-Jun Wu

**Affiliations:** Genetic Lab, Rice Research Institute, Sichuan Agricultural UniversityYa'an, China

**Keywords:** rice, WGD, DNA methylation, gene expression, small RNA

## Abstract

Whole genome duplication (WGD) is a major force in angiosperm evolution. Whether WGD is accompanied by the evolution of epigenetic regulators remains to be explored. Here we investigate whole genome methylation, gene expression, and miRNA regulation among monoploid, diploid, and triploid rice plants isolated from a twin-seedling population. The DNA methylation patterns in the three different ploidy plants were highly similar, with DNA methylation primarily enriched in the promoters. We examined the methylation of single genes and detected around 25,500 methylated genes, of which 22,751 were methylated in all three lines. Significantly divergent DNA methylation patterns between each pair of three lines were only detected in 64 genes, though more genes were found to exhibit differential expression. Analysis of DNA methylation and expression patterns showed that higher DNA methylation levels upstream of the transcription start sites are correlated with higher levels of expression of related genes; whereas higher DNA methylation levels in gene body regions are correlated with lower levels of expression. We also carried out high-throughput sequencing of small RNA libraries and identified 36 new miRNAs. These miRNAs have different expression levels depending on the ploidy.

## Introduction

Polyploidy, including autopolyploidy, is an important force in plant evolution (Wendel, 2000; Ma et al., 2004). In the process of genome doubling, there are a number of adaptations, such as epigenetic changes through methylation and retrotransposon insertion, and these can result in non-functionalization, neo-functionalization and sub-functionalization of genes (Ma and Bennetzen, 2004; Ma et al., 2004). While most research has focused on studying polyploidy in arabidopsis, oilseed rape, cotton, maize, and wheat, there has been limited investigation of polyploidy in rice.

Twin- and triplet-seedling is a phenomenon in which two and three shoots arise from a single seed, respectively. The production of more than one seedling per seed is generally rare in most plants, including rice. Twin- or triplet-seedling rice may exist in many forms, such as monoploid-diploid, diploid-diploid, diploid-triploid, diploid-tetraploid, monoploid-diploid-triploid and so on. Although these plants (with distinct ploidy levels) are genetically identical, the somatic cells carry different DNA copy numbers, which may have important ramifications for gene expression and regulation and may subsequently lead to significant, noticeable phenotypic variations, e.g., variations in plant height, seed production and leaf area. However, the underlying mechanisms for these phenotypic differences remain poorly understood.

SARII-628 is a twin-seedling strain bred from the Rice Research Institute of Sichuan Agricultural University. In the natural population, it normally emerges as a twin-seedling plant with different ploidy, including monoploid and diploid (1N: 2N), and diploid and triploid (2N: 3N). The offspring is stable and can become an earlier germinating line if triploid female are crossed with ordinary diploid male plants. Stable early generations are maintained through further crossing of the resulting diploid individuals with ordinary diploid lines. This phenomenon has important practical significance, enabling shortening of the breeding period.

Recent studies have shown that increased ploidy levels in plants lead to many heritable and phenotypic changes, which do not follow the classical rules of mendelian genetics. The ploidy levels are not linearly correlated with such variation (Doyle et al., 2008; Soltis et al., 2009). Heritable epigenetic mechanisms such as DNA methylation, small RNA regulation and histone modifications play important roles in phenotypic variation (Chen, 2007; Paszkowski and Grossniklaus, 2011). It is plausible that epigenetic variation has been involved in the processes of evolution and speciation (Rapp and Wendel, 2005).

Epigenetic differences have been implicated as a critical factor underlying phenotypic variations (Pikaard, 2001; Adams and Wendel, 2005). DNA methylation is an important epigenetic modification involved in the regulation of gene expression and the control of genome stability in higher eukaryotes. DNA methylation occurs through the addition of a methyl group to the cytosine residue of DNA molecules. In plants, methylation predominantly occurs at CpG, CpHpG, and CpHpH sites, where H represents any nucleotide but guanine. DNA methylation is involved in diverse biological processes, such as transposon silencing, regulation of gene expression, gene imprinting and heterosis (Lippman et al., 2004; Zilberman et al., 2007; Groszmann et al., 2011; Shen et al., 2012). Moreover, the methylation level in rice is four times higher than that in *Arabidopsis thaliana* (Li et al., 2012). While extensive methylation is present in heterochromatic regions of DNA, it is also found in euchromatic regions, including transcribed regions of genes (Zhang et al., 2006; Zilberman et al., 2007). DNA methylation in promoter regions represses gene expression, whereas methylation in gene bodies appears to be positively correlated with gene expression in plants (Cokus et al., 2008; Lister et al., 2008; Li et al., 2012). Importantly, at the genome level a major determinant of differences in methylation patterns is DNA sequence divergence (Li et al., 2012; Schubeler, 2012). Several methods have been developed for genome-wide DNA methylation or methylome profiling. These methods can be classified into three main categories, (i) restriction enzyme-based methods, (ii) bisulfite conversion-based approaches, and (iii) methods using 5-methylcytosine-specific antibodies (methylated DNA immunoprecipitation, or MeDIP), each of which can be combined with microarray analysis or next-generation sequencing (NGS) to determine methylation patterns along the genome (Beck and Rakyan, 2008; Lister and Ecker, 2009; Laird, 2010). While these methods have advantages and disadvantages in terms of accuracy, resolution, complexity and cost, MeDIP followed by NGS, i.e., MeDIP-seq, is perhaps the most cost-effective and has therefore been widely adopted for genome-wide methylome profiling (Down et al., 2008).

MicroRNA or miRNAs are a subset of endogenously initiated, single stranded non-coding RNA, is another important epigenetic regulator involved in the regulation of gene expression by both transcriptional and posttranscriptional mechanisms in a wide variety of eukaryotic organisms (Carrington and Ambros, 2003; Carthew and Sontheimer, 2009). A lot of research found that the majority of miRNAs have a negative regulation on the target mRNA (Nilsen, 2007). High-throughput sequencing to profile small RNAs is a useful tool to identify new miRNAs.

To explore the possible connection among epigenetic regulators through methylation, miRNA and phenotypic variations in rice lines of various ploidy levels, we screened a population of rice (*Oryza sativa*) that has a low frequency of twin- and triplet-seedlings, identified monoploid, diploid and triploid rice plants and profiled their methylomes using MeDIP-seq, along with profiling of small non-coding RNAs. The results of this study provide important insights into differences in epigenetic patterns in rice lines with different ploidy levels.

## Materials and methods

### Methyl-DNA immunoprecipitation and illumina genome analyzer sequencing

SARII-628 autotriploid, monoploid, and the corresponding diploid rice plants derived via natural genesis from twin-seedlings were employed as research materials in this study. After homologous and ploidy identification, the top leaves were cut off the plants and the ratoons were transplanted in a greenhouse and maintained under consistent watering and fertilization conditions. Total genomic DNA was extracted from flag leaves at the meiosis stage using a Plant Dneasy Mini Kit (QIAGEN, US). After quantification with a spectrophotometer, the genomic DNA was sonicated to produce random fragments ranging in size from 200 to 600 bp. A sample from each of the three plants containing 4 mg of fragmented DNA was sent to the Beijing Genomics Institute (BGI) for standard MeDIP analysis as described (Pomraning et al., 2009).

### Public data and MeDIP-Seq sequence alignments

The rice reference genome (*Oryza sativa* L. ssp. *japonica*), together with the reference gene sequences and annotation for repeats and genes, were downloaded from a public database (http://rice.genomics.org.cn/rice/link/download.jsp). The 50 bp MeDIP-Seq reads were aligned to the rice reference genome using the open-source aligner Mapping; 2 bp mismatches were allowed, and uniquely mapped reads were subjected to further analysis.

### Digital gene expression profile (DGE) library construction and sequencing

Total RNA was extracted from the same tissues and at the same developmental stage as was used for MeDIP-seq using an RNeasy Kit, followed by DNaseI treatment. The quality and quantity of RNA were examined by gel electrophoresis and spectrophotometry. Three DGE libraries were constructed from total RNA from monoploid, diploid and triploid plants. Total RNA from the three samples was incubated with magnetic oligo (dT) beads. First- and second-strand cDNA was synthesized and then digested with the restriction enzyme NlaIII, digesting 3′ of CATG sites. Subsequently, the digested cDNA was ligated to the Illumina adapter 1, containing an MmeI restriction site, which digests DNA 20 bp downstream of TCCRAC sites. Following digestion with MmeI the adapter 1 ligated tags were detached from the beads and the Illumina adapter 2 was ligated to the 3′ end. PCR amplification with 15 cycles was performed using primers complementary to the adapter sequences. The resulting 50 bp fragments were purified by PAGE. The DNA was eluted, precipitated, resuspended in 10 μl of 10 mM Tris-HCl (pH 8.5) and quantified by spectrophotometry. The single-stranded products were fixed to an Illumina sequencing chip for sequencing, generating raw reads 50 bp in length. After the raw data was filtered to remove the tag sequences, mapping to the Tigr6.1 (*japonica*) database was performed with the BWA algorithm (**Table 2**), allowing 1 bp mismatches. Uniquely mapped reads were retained for further analysis. Using the DGE method, which generates absolute rather than relative gene expression measurements and avoids many of the inherent limitations of microarray analysis, variations in gene expression in the homologous monoploid, diploid and triploid samples were detected.

### sRNA library construction and sequencing

The middle parts of flag leaves were collected for RNA extraction at 08:00 on the day when the flag leaf was fully extended (as determined by recording the length of the flag leaf each morning until this value no longer increased). Total RNA was isolated from the samples with Trizol Reagent (Invitrogen, USA, Cat. No.18068) following the manufacturer's instructions. Equivalent amounts of total RNA from three biological replicates were sampled and mixed together for the construction of monoploid, autotriploid, and diploid small RNA libraries. The small RNA libraries were sequenced using an Illumina Genome Analyzer, as described by the manufacturer. To validate target cleavage, a modifiedprocedure for 5′-RACE was performedusing the GeneRacer™ kit (Invitrogen) as described.

## Results

### Phenotypic differences among monoploid, diploid, and triploid rice

**Figure 1:**
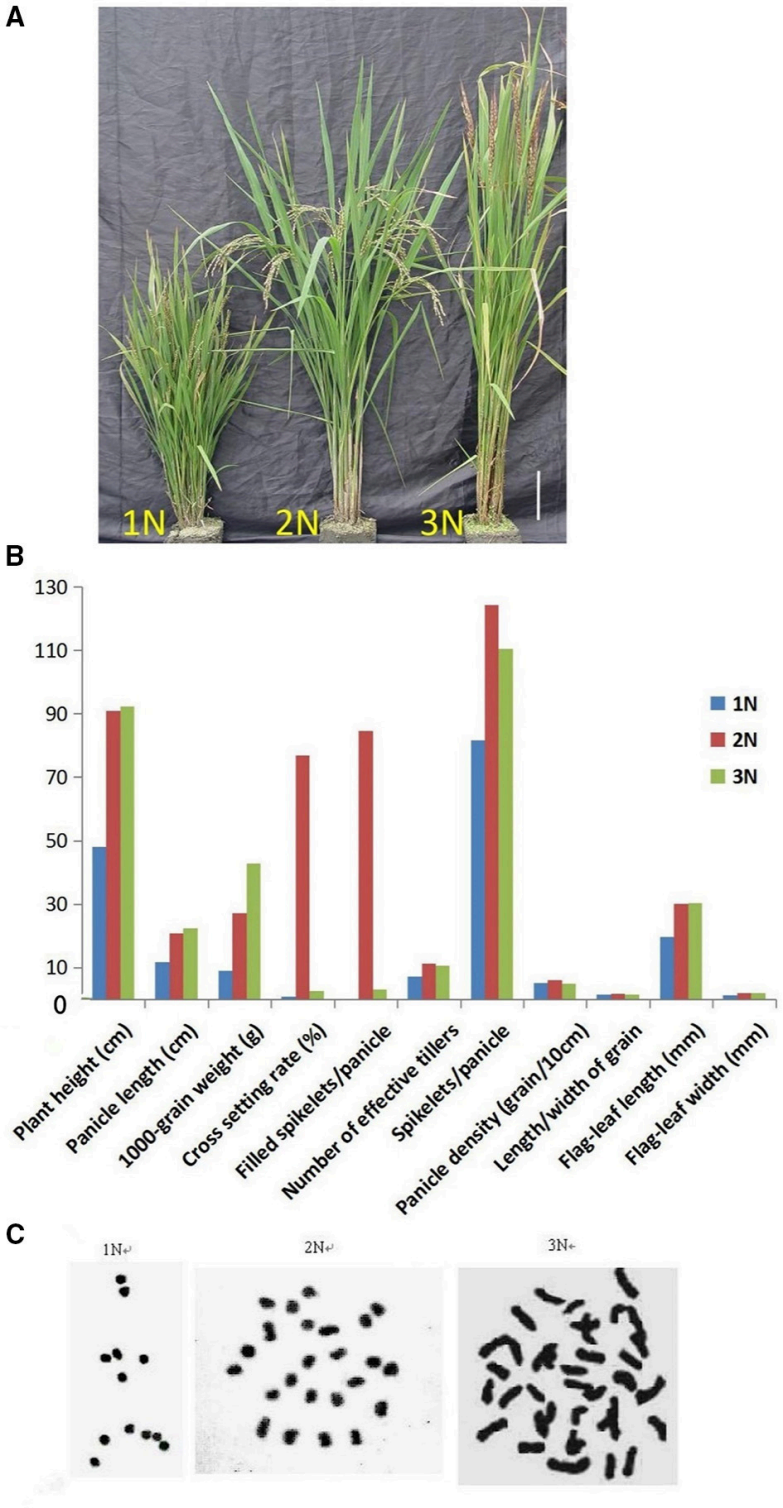
**(A)** Comparison of the phenotypes of monoploid (1N), diploid (2N), and triploid (3N) rice plants grown in the field. **(B)** Phenotypic characteristics of monoploid (1N), diploid (2N), and triploid (3N) rice. **(C)** Chromosome numbers in root tip cells in rice plants with different ploidy levels. There were 12 chromosomes in 1N rice, 24 chromosomes in 2N rice and 36 chromosomes in 3N rice.

The SARII-628 line can segregate the monoploid, diploid and triploid plants at very low frequency (2–5%) from the natural population. These three plant lines exhibited different phenotypes. The monoploid rice was dwarfed and completely sterile, with thin, short leaves, and small glumes. The diploid rice had short and partial awns but normal seed, while the triploid rice was tall and sterile, with large organs and long awns (Figures 1A,B). As expected, these rice plants carried the characteristic numbers of chromosomes, monoploid, diploid, and triploid plants having 12, 24, and 36 chromosomes respectively (Figure 1C). Despite the distinct numbers of chromosomes, we detected 192 Simple Sequence Repeats (SSR) in the three different rice ploidy lines and found there were no differences at the same sites within these lines. This suggested these rice lines harbor the same genetic information but at different ploidy levels.

### The three rice lines have similar methylomes

**Figure 2:**
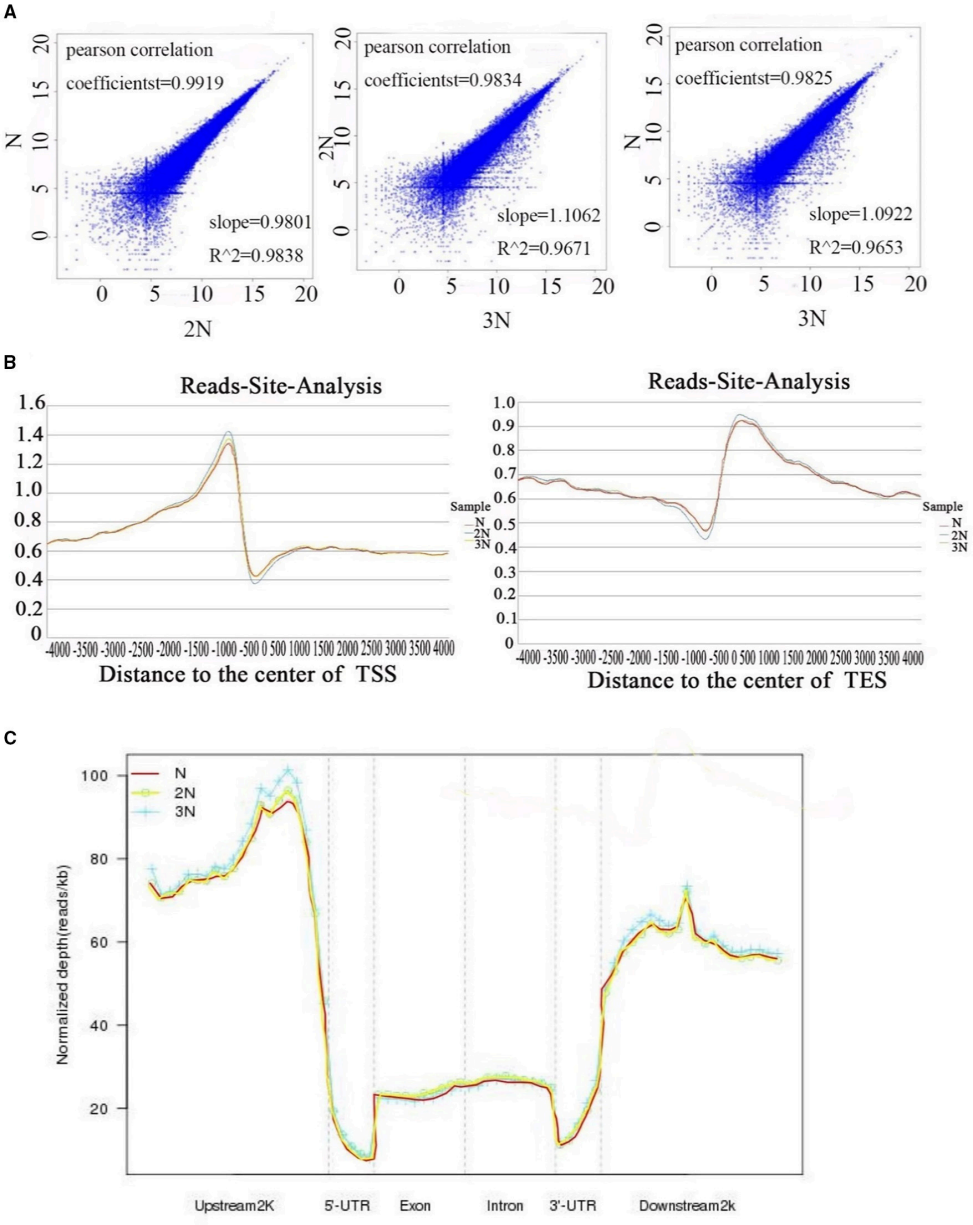
**(A)** Correlation of methylation levels in gene body regions between two different libraries of 1N, 2N, and 3N rice. The X-axis and Y-axis show the normalized number of reads for gene body regions in each sample. ZHY_N_body, ZHY_2N_body, ZHY_3N_body represent monoploid, diploid and triploid gene body regions, respectively. **(B)** Methylation reads distribution in gene body and flanking regions. **(C)** Peak distribution in different genomic regions. Methylation density is the ratio of methylation peaks to the corresponding sequence lengths.

We addressed the question whether the methylation pattern will be affected when genome duplication occurs. The methylation patterns of the monoploid, diploid and triploid plants were analyzed using MeDIP-seq (see Materials and Methods). Initially the methylomes of the three rice lines were examined at the chromosome level at a resolution of 10 kb (Supplementary Figure 1). Taking the sequencing depths into consideration, the DNA methylation levels in the monoploid, diploid and triploid rice, as normalized by the sequence lengths, exhibited a 1:2:3 ratios, respectively. A total of 498 million raw reads were generated for the three samples, and more than 50% of the reads in each sample uniquely mapped to the rice genome (Table 1). Uniquely mapped reads distributed in gene bodies were used for association analysis between any two samples from different ploidy lines, as well as between 2N rice, and the results of analysis of another rice line by He et al. (2010). The results indicated that the DNA methylation patterns of the three types of plants were highly conserved, as 98.25 to 99.19% of their corresponding methylated sites were correlated (Figure 2A). Note that the observed distribution of methylation patterns was partially consistent with the results of He et al. (2010), as the distribution of methylated reads from the two studies exhibited a 63.48% correlation (Supplementary Figure 2).

**Table 1:** **Statistics of the reads from MeDIP-Seq, RNA-seq, and small RNA-seq of monoploid, diploid, and triploid rice**.

**Reads category**	**MeDIP-seq**			**RNA-seq**			**Small RNA-seq**		
	**1N**	**2N**	**3N**	**1N**	**2N**	**3N**	**1N**	**2N**	**3N**
Raw reads	48.98M	48.98M	48.98M	8.08 M	8.19M	7.86M	7.21M	7.11M	6.20M
Mapped reads	42.16M (86.1%)	42.26M (86.3%)	42.17M (86.1%)	4.34M (53.8%)	4.21M (51.5%)	4.50M (57.2%)	5.00M (69.2%)	5.18M (72.8%)	4.37M (70.5%)
Uniquely mapped	21.33M (50.6%)	21.34M (50.5%)	21.64M (51.3%)	3.53M (81.3%)	3.50M (83.1%)	3.63M (80.7%)	1.50M (30.0%)	1.13M (21.8%)	1.21M (27.7%)

M, million.

The uniquely mapped reads were also used to detect methylation peaks (*p* < 1e^−5^) of genomic regions. In total, we detected 67,307, 65,208, and 65,932 peaks, ranging from 200 to 5,475 bp long, in the genomes of monoploid, diploid and triploid rice, respectively; these peaks covered ~15% of the genomes (Table 2). To further investigate the methylation distribution characteristics in the genomes, we overlapped all reads of the peaks that mapped to a region 4 kb upstream and 4 kb downstream of the transcription start sites (TSS) and transcription end sites (TES). The results revealed that methylation was enriched within a 1.5 kb region upstream of the TSS and within a 1.5 kb region downstream of the TES (Figure 2B). Furthermore, to assess the methylation patterns in the genomes, we classified the methylation peaks into six groups: promoter, intron, exon, 5′ untranslated region (5′UTR), 3′ untranslated region (3′UTR) and downstream 2 kb. We also normalized the proportion of methylation peaks of each class by length with respect to the lengths of their corresponding genomes. The promoter and downstream 2kb regions were hypermethylated (Li et al., 2008), whereas the 5′UTRs were hypomethylated (low read density; Figure 2C). We found that in all three rice ploidy lines, the methylation sites were distributed in different genomic regions in a similar pattern, including coding and noncoding regions. Together, these results indicate that the distribution patterns of methylated sites in the three rice lines were similar at the genome level, suggesting that the effects of variations in ploidy on methylation may be localized to different functional regions of the genome.

**Table 2:** **Information about the methylation peaks determined through MeDIP-seq**.

**Sample**	**Peak numbers**	**Peak average length (bp)**	**Peaks total length (bp)**	**Peaks coverage (%)**	**Gene numbers involved**
1N	67307	1417.14	58.27M	15.65	25653
2N	65208	1408.35	57.88M	15.55	25103
3N	65932	1254.45	57.09M	15.33	25285

M, million.

We found that the methylation profiles of three rice lines with different ploidy levels were similar at the chromosome scale, and the distribution of methylation did not significantly differ in gene bodies, promoters and intergenic regions among 1N, 2N, and 3N rice. These results are similar to the findings of a recent methylome study of an autotetraploid (4X) rice line and its diploid donor, cultivar Aijiaonante (2X) (Zhang et al., 2015). Together, these findings suggest that ploidy may not have a widespread influence over DNA methylation.

### Variations in methylation at the genic level

**Figure 3:**
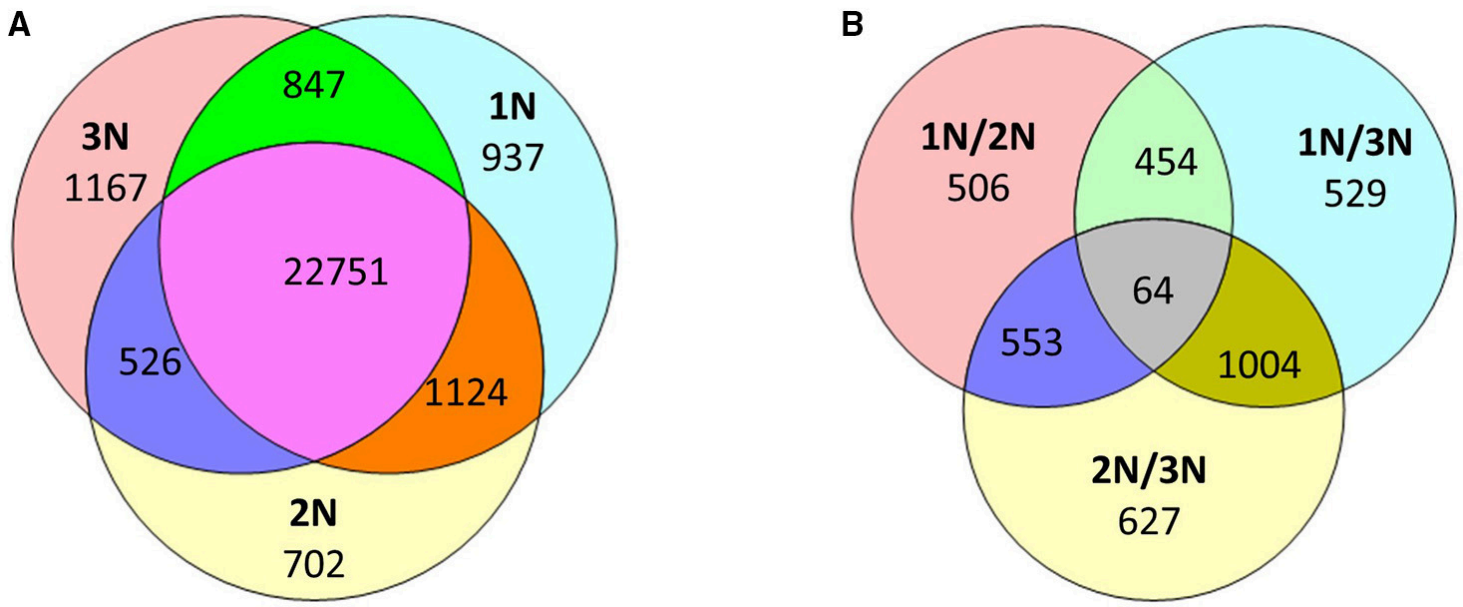
**(A)** Number of methylated genes that were unique or shared among the three rice lines (1N is monoploid, 2N is diploid, and 3N is triploid). **(B)** Number of differentially methylated genes among the three rice lines and between any two rice lines.

While the methylomes of the three rice plants did not appear to differ significantly when examined at a low resolution, a substantial number of genomic loci with variations in methylation patterns and levels were observed across the three rice lines. For convenience, genes with methylation peaks within the region 1.5 kb upstream of the TSS to the TES of each gene are hereafter referred to as methylated genes. In total, 28,048 methylated genes were found in three rice lines: 25,653 in monoploid rice, 25,103 in diploid rice and 25,285 in triploid rice (Supplementary Table 1). Of these, 937 (3.6% of the total), 702 (2.8%), and 1167 (4.6%) genes were methylated exclusively in monoploid, diploid, and triploid rice, respectively, and 22,751 genes were methylated in all three lines (Figure 3A).

To assess variations in the methylome, we compared methylation levels across pairs of plants with different ploidy levels. A total of 3737 genes exhibited at least two-fold differences in methylation levels between any two rice lines with different ploidy levels. These genes included 1047 TE genes, 1250 hypothetical genes, and 1440 protein-coding genes. In particular, 506, 529, and 627 genes were differentially methylated exclusively between the monoploid and diploid, monoploid and triploid and diploid and triploid plants, constituting 13.5, 14.2, and 16.8% of the 3737 differentially methylated genes, respectively (Figure 3B). In addition, 1577 and 2248 genes were differentially methylated with increasing ploidy. The highest fold change in methylation was 28-fold. These results suggest that as ploidy changes, methylation patterns and methylation levels change. Consistent with previous findings, approximately 40% of gene sites in rice were methylated (Li et al., 2008; Yan et al., 2010), 16% of which had significantly different methylation levels between at least two lines of different ploidy levels. These differentially methylated genes included not only TE sequences, but also protein-coding sequences. However, Zhang et al. reported that a diploid and autotetraploid rice line only exhibited variations in methylation patterns in transposable elements regions (Zhang et al., 2015). This discrepancy may be related to the observation that, in the current study, the same genes in 1N, 2N, and 3N rice sometimes exhibited differential expression patterns, whereas Zhang et al. did not detect significant differences in the expression of the majority of the genes between autotetraploid rice and its diploid donor.

To gain insight into the potential functions of methylation, we analyzed variations in the methylation patterns of the rice lines. In particular, we identified genes whose methylation levels changed as the degree of ploidy increased. By lowering the standard to a 1.5 fold change, we identified 87 and 150 genes with increased and reduced methylation levels with increased ploidy, respectively (Supplementary Table 2). The genes with upregulated methylation were significantly enriched in the Gene Ontology (GO) categories of lithium ion transport and various ion homeostasis, whereas the genes with downregulated methylation were enriched in the GO categories RNA-dependent DNA replication, endosperm development, histone methylation, and nucleosome assembly, among many others (Supplementary Table 3). These results indicate that these differentially methylated genes were strongly related to metabolism and stress responses. Ion transport and homeostasis are primarily stress-related (Niu et al., 1995; Chaves-Sanjuan et al., 2014), suggesting that these genes are upregulated during stress responses, correlating with the increased methylation detected in the triploid plants. These functions are related to plant growth and metabolism, suggesting that these genes may be involved in the increase in the organ size in response to the reduced methylation levels as the ploidy increases.

### Effects of DNA methylation on gene expression

DNA methylation in promoter regions and gene bodies is associated with gene expression (Li et al., 2012). To gain insight into the relationship between DNA methylation and gene expression in rice with various ploidy levels, we obtained the digital gene expression (DGE) profiles for the three rice lines using NGS. After filtering low quality reads, we mapped the sequences to the Tigr6.1 (*japonica*) database with the BWA algorithm (Table 3). Only the uniquely mapped reads were used in subsequent analysis. In total, we obtained expression data for 26,667 genes.

**Table 3:** **Number of genes with differential DNA methylation and expression levels in each comparison**.

**DNA methylation levels**	**High**		**Low**	
**Gene expression regulated**	**Up**	**Down**	**Up**	**Down**
1N vs. 2N	1	1	3	0
2N vs. 3N	13	14	24	17
1N vs. 3N	26	4	47	12

Based on the DGE reads, some genes with significantly differential expression with increasing ploidy were screened with the DE-seq algorithm. A total of 295 genes were upregulated and 794 were downregulated with increasing ploidy. We subjected these genes to GO analysis (*P* < 0.01; Supplementary Table 4), finding that the upregulated genes were involved in defense response, acetyl-CoA biosynthetic, systemic acquired resistance, DNA repair and so on. By contrast, the downregulated genes were involved in stress responses, signal transduction, actin activation, ion transport, organism defense, metabolite process and so on.

A summary of the number of genes with significantly different methylation and expression patterns between any two rice lines of different ploidy levels is shown in Table 4. Among these genes, only one gene, LOC_Os06g11330, exhibited increasing methylation and reduced expression (at least two-fold) with increasing ploidy level. LOC_Os06g11330 encodes MADS-box transcription factor 55 and is predicted to be the target of microRNA529a. This gene is an early senescence-associated gene that is expressed in rice flag leaves (Liu et al., 2008). Two genes, LOC_Os09g15380 (encoding a retrotransposon protein) and LOC_Os05g06980 (encoding a hypothetical protein), exhibited decreasing methylation and increasing expression with increasing ploidy level.

**Table 4:** **The annotation of target gene**.

**miRNAs**	**Target**	**Funtion**
Osa-miR9	LOC_Os04g58610	Retrotransposon protein, putative, unclassified, expressed
	LOC_Os01g50860.1	Chloride transporter, chloride channel family, putative, expressed
Osa-miR25	LOC_Os09g25430.1	ZOS9-07—C2H2 zinc finger protein, expressed
Osa-miR31	LOC_Os06g06600.1	OsFBX187—F-box domain containing protein, expressed
Osa-miR827a	LOC_Os04g48390	Uncharacterized membrane protein, putative, expressed

**Figure 4:**
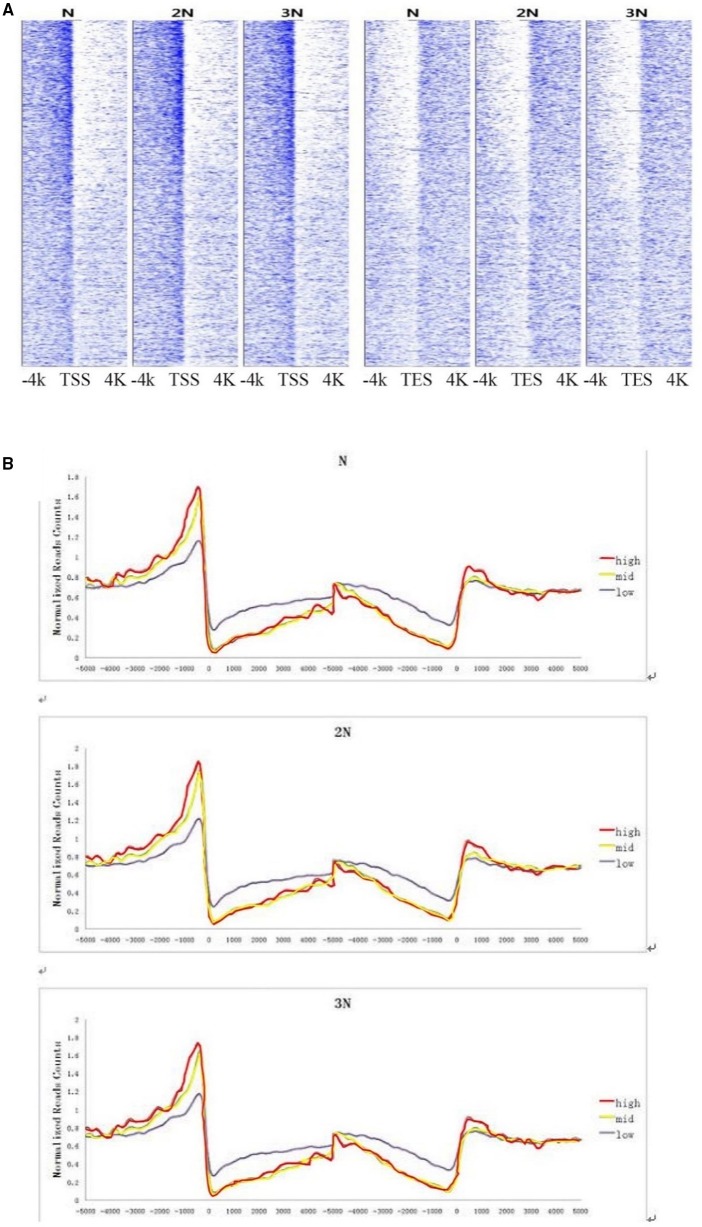
**(A)** Correlation between DNA methylation and gene expression levels at sites 4 kb upstream and downstream of the TSS and TES at the genome-wide level. The X-axis shows the location 4 kb upstream and downstream of the TSS and TES. The Y-axis shows gene expression levels. Each dot represents one DNA methylation read. **(B)** DNA methylation levels of highly expressed, moderately expressed and weakly expressed gene populations. Highly expressed genes: read counts >200; moderately expressed genes: read counts 30–200 (Wendel); weakly expressed genes: read counts <30.

Because there are significant differences of DNA methylation level in two flanking regions of TSS and TES (Figure 2B), we performed genome-wide analysis of DGE reads and methylation peaks in all gene sites in these samples. The results show that the methylation levels exhibited a strong, positive correlation with gene expression levels in regions upstream of TSS regions and a (smaller) negative correlation in regions downstream of TSS and upstream of TES regions. This trend was observed in all three rice lines (Figures 4A,B). Many studies have suggested that gene body methylation is associated with active transcription in *Arabidopsis* (Zhang et al., 2006; Zilberman et al., 2007; Takuno and Gaut, 2013). In rice, Li et al. showed that gene expression appears to be repressed by DNA methylation (Li et al., 2008). He et al. detected a weak negative correlation between DNA methylation and transcript levels (He et al., 2010). Wang et al. suggested that intermediate body methylation tends to be associated with high levels of gene expression, whereas heavy body methylation is associated with lower levels of gene expression (Wang et al., 2013). In the current study, we found that DNA methylation in the promoter region was positively associated with gene expression, but there was a less negative correlation between DNA methylation and gene expression in gene body regions in rice plants of various ploidy levels.

### miRNA function in different ploidy levels

In the present study, we performed high-throughput sequencing of small RNA libraries from three different rice ploidy lines. By blasting the sequences against miRBase V12.0 (Supplementary Table 5) we identified 36 new candidate miRNAs. miRNAs, identified as having an excess of 50 reads, were analyzed for their respective expression levels. A large number of miRNA targeted genes showed strong increased or decreased expression levels in different tissues in the three ploidy lines (unpublished data). We identified 13 miRNAs with altered expression level in the leaves (Supplementary Table 6). All 13 miRNAs showed increased expression levels in monoploid compared to diploid plants. The expression level of 4 miRNAs were shown to be increased in triploid compared to diploid plants, whilst the others showed diminishing levels. Small RNAs have a variety of important roles in plant development such as guiding mRNA cleavage, translational repression, and chromatin modification. Hence, changes in miRNA expression can be expected to influence the expression of targeted genes.

**Figure 5:**
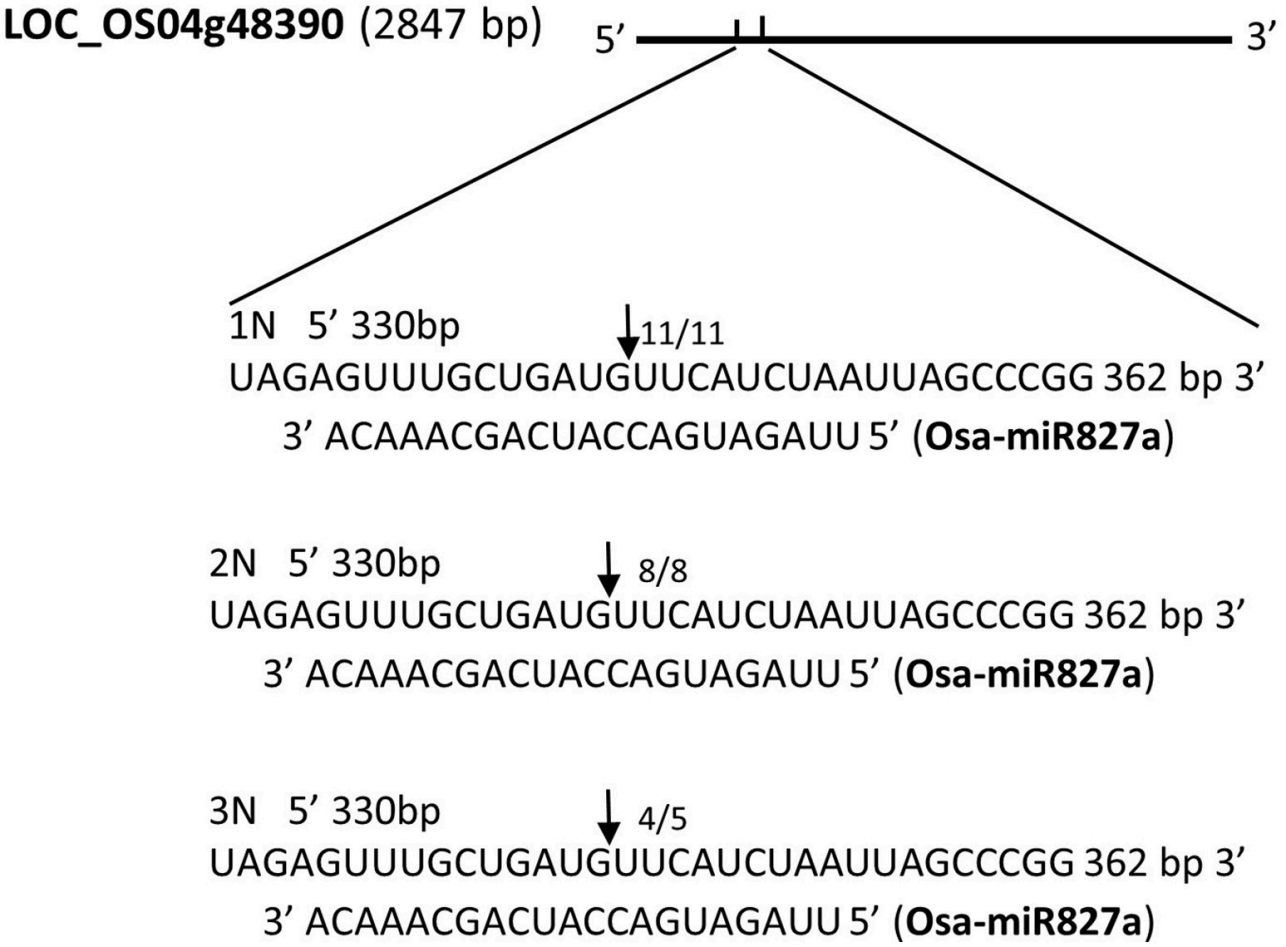
**The cleavage sites of four miRNAs targets by 5′-RACE in monoploid (1N), diploid (2N), and triploid (3N) rice**.

Next, we sought to examine whether mRNA cleavage is also affected by the level of ploidy. We chose randomly four miRNAs, namely, Osa-miR9, Osa-miR25, Osa-miR31, and Osa-miR827a, to investigate miRNA cleavage. Using 5′-rapid amplification of cDNA (5′-RACE), we found different patterns of mRNA cleavage. The first pattern involved cleavage at the same site in the three rice ploidy lines. A target gene of Osa-miR827a is LOC_Os04g48390. The cleavage position, guided by Osa-miR827a, is located between base pairs 10–11 of the target site (A_10_ and A_11_), matching the predicted site of mRNA cleavage. The frequencies of this cleavage position is 11/11, 8/8, 4/5 among the monoploid, diploid, triploid plant respectively (Table 4, Figure 5). The other miRNAs (Osa-miR9, Osa-miR25, and Osa-miR31) showed variable cleavage within a defined locus across the three ploidy lines. Osa-miR9 and Osa-miR31 showed different cleavage sites between all three ploidy lines (Supplementary Figures 3A–B), whilst Osa-miR25 showed identical cleavage sites in the monoploid and diploid lines, 6/6 and 5/5, respectively, and 3 different cleavage sites in the triploid line (Supplementary Figure 3C). Interestingly, many of the target sites were outside the predicted sites for miRNA binding. These results are consistent with those of Lacombe using 5′-RACE (Supplementary Figure 4).

miRNAs target gene expression by associating with an Argonaute (AGO) protein to form an RNA-induced silencing complex (RISC; Vaucheret, 2008). In our study, we investigated 36 miRNAs and found that most of them have higher miRNA expression patterns in monoploid but lower miRNA expression patterns in triploid rice compared to diploid rice. Interestingly, although both monoploid and triploid rice are sterile, they still have big difference in the plant height and seed size. This difference in phenotype may be associated to the differential expression and targeting by miRNAs in these lines.

## Discussion

In this study, we analyzed whole genome DNA methylation patterns, expression patterns and the miRNA profiling in monoploid, diploid and triploid rice, which segregated from double-seedling rice. We found the methylation patterns in the whole genome are the same among the three ploidy lines, which harbor the same genetic information. This observation is consistent with previous studies showing whole genome DNA methylation patterns are stably inherited from parents over generations (Liu and Wendel, 2003). Other reports suggest allopolyploidy associated changes are the outcome of altered gene expression due to various causes, including increased variation in gene expression and rapid genetic and epigenetic changes, probably conferred by genome-wide interactions (Osborn et al., 2003). Similarly with this finding, we identified thousands of genes with altered genes expression levels through transcriptional silencing mechanisms including DNA methylation and miRNA cleavage, and these changes may be correlated with the different phenotypes exhibited by the three ploidy rice lines. Our studies support the idea that changes in whole-genome methylation are not widespread with changes in ploidy level, but altered expression and targeting of miRNAs resulting from genome duplication and altered ploidy levels may be the factors that drive evolution.

## Author contributions

XJW and HYZ designed the study. HYZ, SHW, XFZ, and HXZ carried out most of the data analysis. HYZ, HXZ, and FH wrote the paper. HYZ and FH made contribution to the editing and proofreading of manuscript. All authors read and approved the final manuscript. HXZ submitted the manuscript.

## Funding

This work was supported by the National Nature Science Foundation of China (No. 31301049 and No.30971618).

### Conflict of interest statement

The authors declare that the research was conducted in the absence of any commercial or financial relationships that could be construed as a potential conflict of interest.

## Supplementary material

Supplementary Figure 1 **Chromosome distribution of reads in 1N, 2N, and 3N rice**. The distribution of reads on each chromosome, including those of 1N, 2N, and 3N rice, is shown.

Supplementary Figure 2 **Correlation between the methylation levels detected in the current study and those of He et al**.

Supplementary Figure 3 **(A–C)** The cleavage sites of three miRNAs targets by 5′-RACE in monoploid (1N), diploid (2N), and triploid (3N) rice.

Supplementary Figure 4 **Lacombe's results of cleavage sites of Osa-miR872a targets determined by 5′-RACE**.

Supplementary Table 1 **Methylated genes in the monoploid, diploid, triploid rice lines**.

Supplementary Table 2 **Deregulated methylation levels with increased ploidy levels**.

Supplementary Table 3 **Enriched Go terms of deregulated methylation levels with increased ploidy (from 1N, 2N to 3N)**. GO terms are sorted by their *P-*value (lower to higher). Only Go categories with a *P* < 0.01 are shown.

Supplementary Table 4 **Enriched Go terms of commonly deregulated genes among monoploid, diploid, triploid rice**. GO terms are sorted by their *P*-value (lower to higher). Only Go categories with a *P* < 0.01 are shown.

Supplementary Table 5 **Novel candidate miRNAs of rice**.

Supplementary Table 6 **Changed expression levels of miRNAs in rice ploidy lines**. The number indicates fold change of expression in monoploid or triploid lines compared to diplioid lines.

## Abbreviations

1N: monoploid
2N: diploid
3N: triploid
TSS: transcription start site
TES: transcription end site.
