# Supplementary figures and images for: Global Methylation Patterns and Their Relationship with Gene Expression and Small RNA in Rice Lines with Different Ploidy
Source: Front Plant Sci. 2016 Jul 21;7:1002. doi: 10.3389/fpls.2016.01002 (PMC4954823; doi:10.3389/fpls.2016.01002)

S1

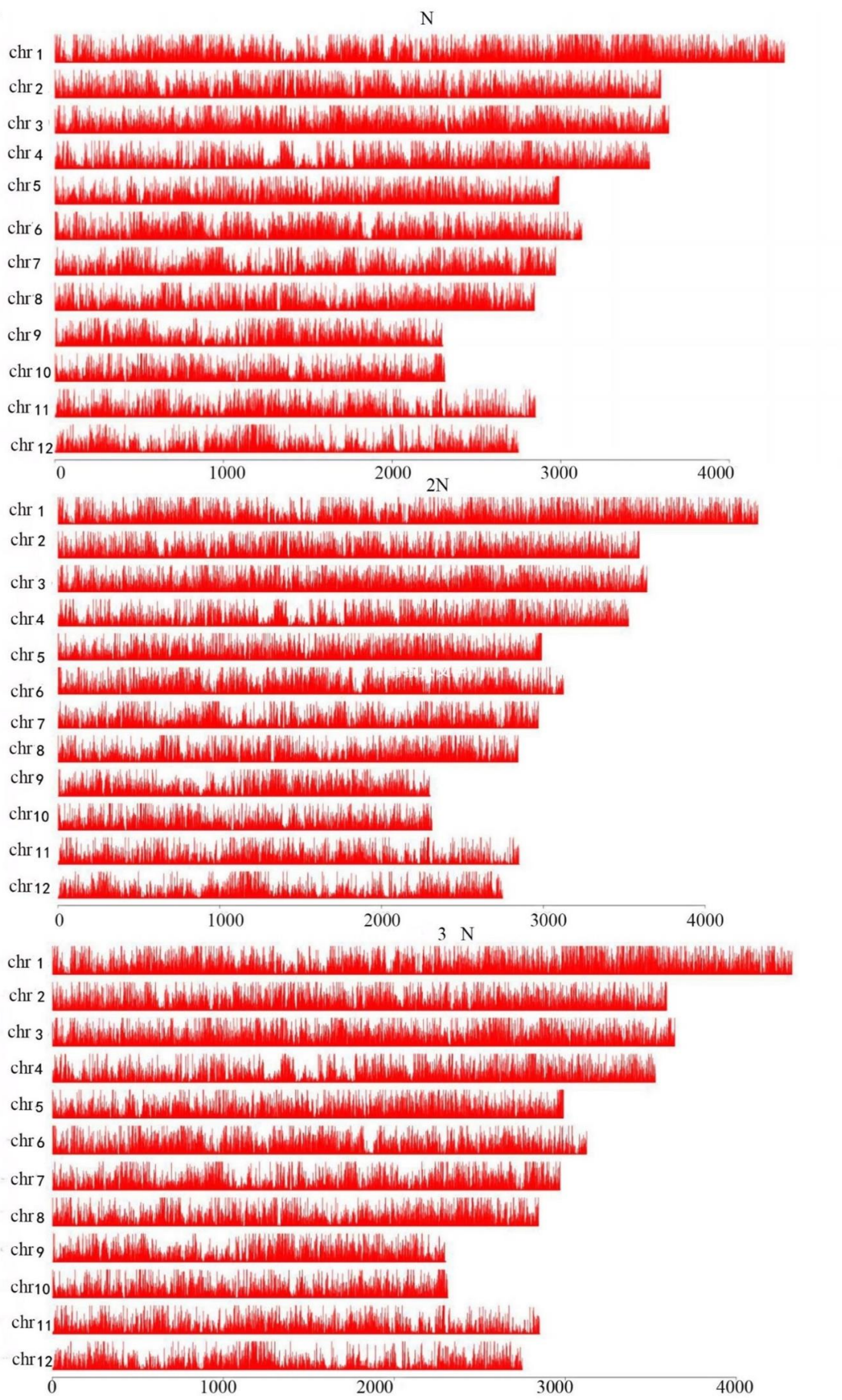

S2

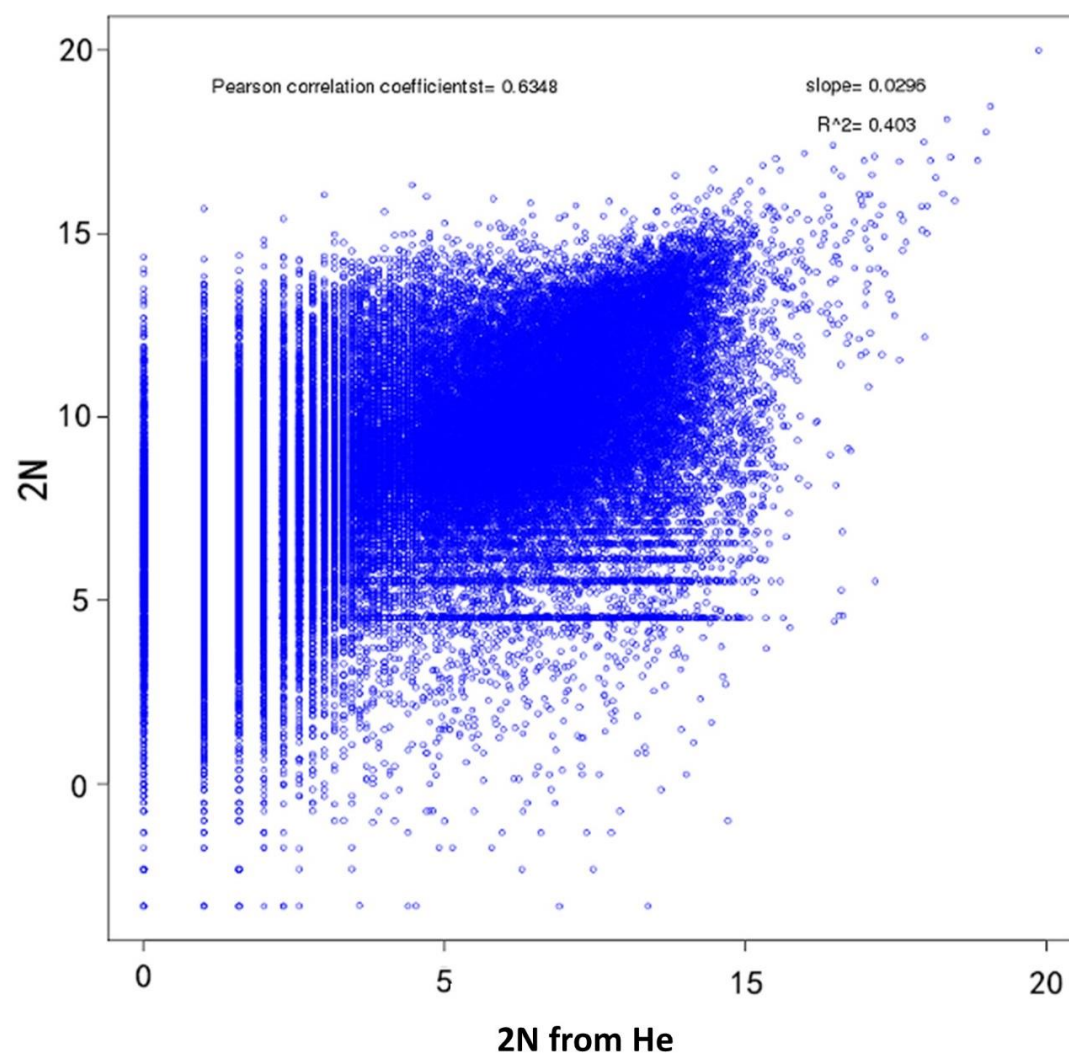

S3

A

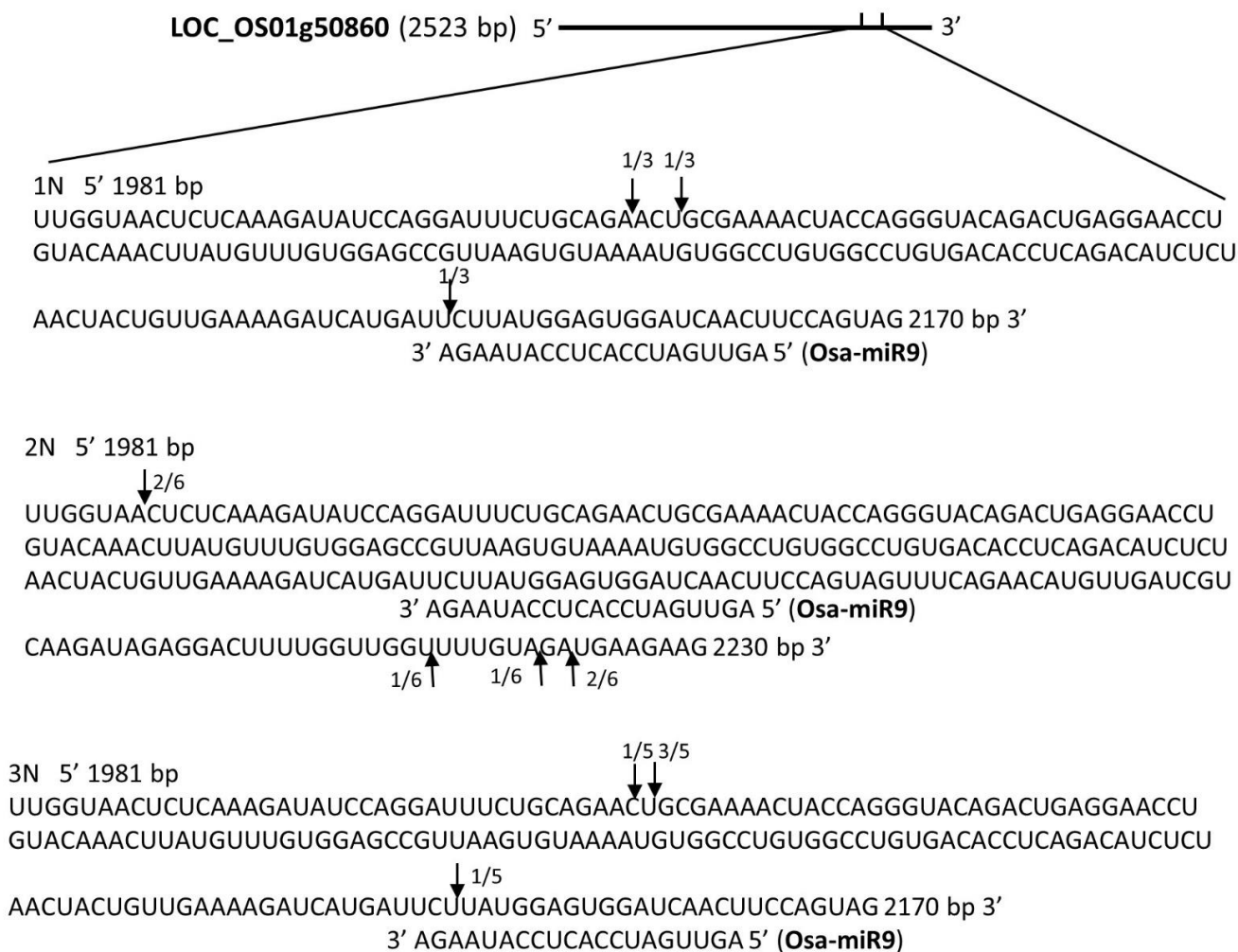

S3

B

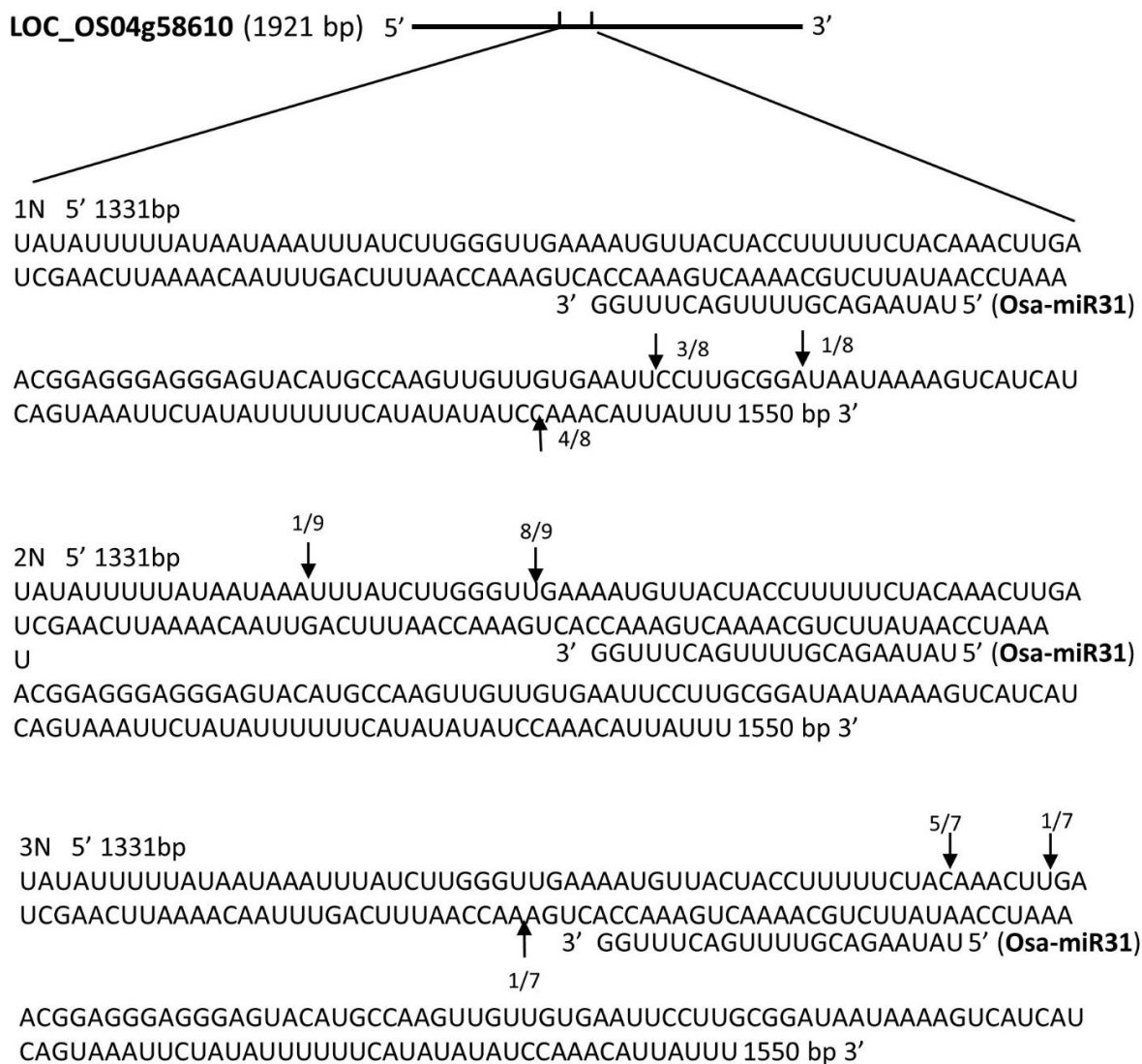

S3

C

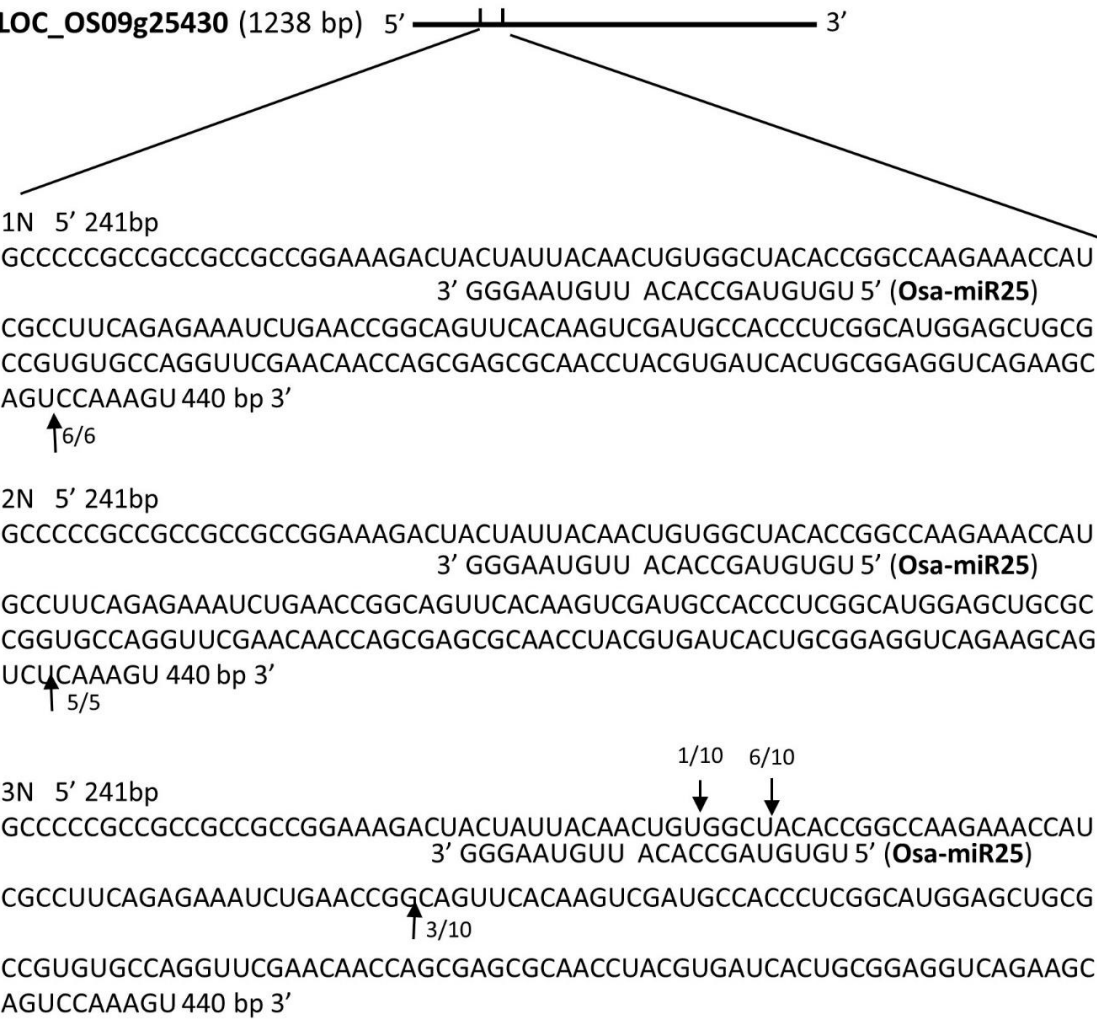

S4

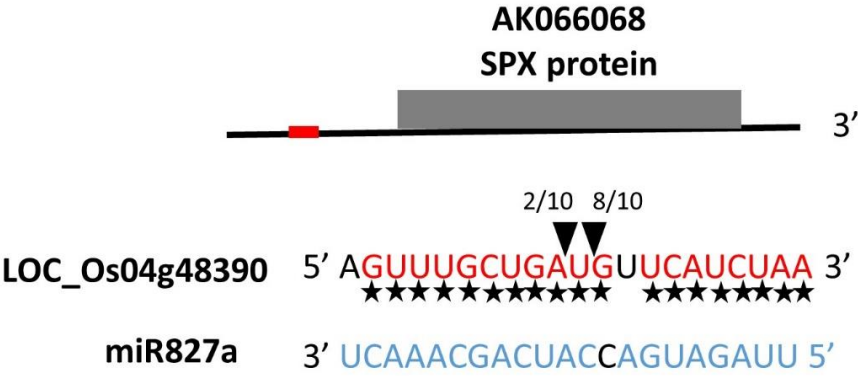

Supplement: Supplementary Figure 1 — Chromosome distribution of reads in 1N, 2N, and 3N rice. The distribution of reads on each chromosome, including those of 1N, 2N, and 3N rice, is shown. [file Image1.pdf]
