# Supplementary material for: Global Methylation Patterns and Their Relationship with Gene Expression and Small RNA in Rice Lines with Different Ploidy
Source: Front Plant Sci. 2016 Jul 21;7:1002. doi: 10.3389/fpls.2016.01002 (PMC4954823; doi:10.3389/fpls.2016.01002)
Supplement: Supplementary Table 1 — Methylated genes in the monoploid, diploid, triploid rice lines. [file DataSheet3.docx]

**Supplementary Table1**

**Methylated genes in the monoploid, diploid, triploid rice lines.**

**Supplementary Table2**

**Deregulated methylation levels with increased ploidy levels.**

**SupplementaryTable3**

**Enriched Go terms of deregulated methylation levels with increased ploidy (from 1N,2N to 3N). GO terms are sorted by their *P -value* (lower to higher). Only Go categories with a *P -value*＜0.01 are shown.**

**Increased methylation levels with increased ploidy**

| **GO Term** | **P-Value** | **FDR** | **Description** |
| --- | --- | --- | --- |
| GO:0010351 | 0.0035 | 0.445714 | lithium ion transport |
| GO:0009639 | 0.0037 | 0.445714 | response to red or far red light |
| GO:0006882 | 0.0087 | 0.445714 | cellular zinc ion homeostasis |
| GO:0015884 | 0.0087 | 0.445714 | folic acid transport |

**Decreased methylation levels with increased ploidy**

| **GO Term** | **P-Value** | **FDR** | **Description** |
| --- | --- | --- | --- |
| GO:0006278 | 0.0035 | 0.645 | RNA-dependent DNA replication |
| GO:2000014 | 0.0043 | 0.645 | regulation of endosperm development |
| GO:0016571 | 0.0075 | 0.67 | histone methylation |
| GO:0006334 | 0.0092 | 0.67 | nucleosome assembly |

**Supplementary Table4**

**Enriched Go terms of commonly deregulated genes among monoploid, diploid, triploid rice. GO terms are sorted by their *P -value* (lower to higher). Only Go categories with a *P -value*＜0.01 are shown.**

**Commonly up -regulated genes among monoploid, diploid, triploid rice**

| **GO Term** | **Numberof genes** | **P-Value** | **FDR** | **Description** |
| --- | --- | --- | --- | --- |
| GO:0009870 | 3 | 0.00089 | 0.114633 | defense response signaling pathway, resistance gene-dependent |
| GO:0006086 | 2 | 0.00186 | 0.114633 | acetyl-CoA biosynthetic process from pyruvate |
| GO:0009627 | 4 | 0.0041 | 0.114633 | systemic acquired resistance |
| GO:0006281 | 7 | 0.00426 | 0.114633 | DNA repair |
| GO:0010565 | 3 | 0.00512 | 0.114633 | regulation of cellular ketone metabolic process |
| GO:0006457 | 8 | 0.00543 | 0.114633 | protein folding |
| GO:0006364 | 4 | 0.00556 | 0.114633 | rRNA processing |
| GO:0000398 | 3 | 0.00584 | 0.114633 | nuclear mRNA splicing, via spliceosome |
| GO:0051016 | 1 | 0.00831 | 0.114633 | barbed-end actin filament capping |
| GO:0046109 | 1 | 0.00831 | 0.114633 | uridine biosynthetic process |
| GO:0080158 | 1 | 0.00831 | 0.114633 | chloroplast ribulose bisphosphate carboxylase complex biogenesis |
| GO:0048481 | 3 | 0.00833 | 0.114633 | ovule development |

**Commonly down -regulated genes amongmonoploid, diploid, triploid rice**

| **GO Term** | **Number of genes** | **P-Value** | **FDR** | **Description** |
| --- | --- | --- | --- | --- |
| GO:0046686 | 27 | 0.000016 | 0.0048 | response to cadmium ion |
| GO:0006200 | 28 | 0.000051 | 0.00765 | ATP catabolic process |
| GO:0009961 | 3 | 0.00028 | 0.0255 | response to 1-aminocyclopropane-1-carboxylic acid |
| GO:0070588 | 6 | 0.00047 | 0.0255 | calcium ion transmembrane transport |
| GO:0055122 | 2 | 0.0006 | 0.0255 | response to very low light intensity stimulus |
| GO:0000291 | 2 | 0.0006 | 0.0255 | nuclear-transcribed mRNA catabolic process, exonucleolytic |
| GO:0007165 | 98 | 0.00064 | 0.0255 | signal transduction |
| GO:0009615 | 10 | 0.00068 | 0.0255 | response to virus |
| GO:0009651 | 34 | 0.00086 | 0.02825 | response to salt stress |
| GO:0030048 | 4 | 0.00105 | 0.02825 | actin filament-based movement |
| GO:0031087 | 3 | 0.00111 | 0.02825 | deadenylation-independent decapping of nuclear-transcribed mRNA |
| GO:0035556 | 17 | 0.00113 | 0.02825 | intracellular signal transduction |
| GO:0009862 | 4 | 0.00155 | 0.035769 | systemic acquired resistance, salicylic acid mediated signaling pathway |
| GO:0015711 | 6 | 0.00205 | 0.0422 | organic anion transport |
| GO:0055081 | 3 | 0.00211 | 0.0422 | anion homeostasis |
| GO:0042742 | 18 | 0.00249 | 0.046688 | defense response to bacterium |
| GO:0006680 | 2 | 0.0035 | 0.058667 | glucosylceramide catabolic process |
| GO:0006536 | 4 | 0.00352 | 0.058667 | glutamate metabolic process |
| GO:0050896 | 281 | 0.00466 | 0.069962 | response to stimulus |
| GO:0009620 | 18 | 0.00501 | 0.069962 | response to fungus |
| GO:0008643 | 11 | 0.00513 | 0.069962 | carbohydrate transport |
| GO:0006355 | 79 | 0.00572 | 0.069962 | regulation of transcription, DNA-dependent |
| GO:0046500 | 2 | 0.00574 | 0.069962 | S-adenosylmethionine metabolic process |
| GO:0040029 | 10 | 0.00586 | 0.069962 | regulation of gene expression, epigenetic |
| GO:0006413 | 7 | 0.00637 | 0.069962 | translational initiation |
| GO:0032312 | 3 | 0.00652 | 0.069962 | regulation of ARF GTPase activity |
| GO:0015996 | 3 | 0.00652 | 0.069962 | chlorophyll catabolic process |
| GO:0009086 | 4 | 0.00672 | 0.069962 | methionine biosynthetic process |
| GO:0006730 | 18 | 0.00744 | 0.069962 | one-carbon metabolic process |
| GO:0009084 | 4 | 0.00754 | 0.069962 | glutamine family amino acid biosynthetic process |
| GO:0006886 | 17 | 0.00769 | 0.069962 | intracellular protein transport |
| GO:0048519 | 26 | 0.00791 | 0.069962 | negative regulation of biological process |
| GO:0010587 | 2 | 0.00848 | 0.069962 | miRNA catabolic process |
| GO:2000022 | 2 | 0.00848 | 0.069962 | regulation of jasmonic acid mediated signaling pathway |
| GO:0007017 | 9 | 0.00863 | 0.069962 | microtubule-based process |

**Supplementary table5:Novel candidate miRNAs of rice**

| **miRNA** | **Sequence(5’-3’)** | **miRNA** | **Sequence(5’-3’)** |
| --- | --- | --- | --- |
| Osa-miR#1 | tcagacttgaagcagtttaac | Osa-miR#27 | aggtcaactgactgtgatggc |
| Osa-miR#2 | aaaatcggtttcggacaagtc | Osa-miR#29 | atagcaacttaggatcgaatggga |
| Osa-miR#3 | caaaattttccatgcacttcga | Osa-miR#30-5p1 | gcgtctgtagtgaatagaaaacgaca |
| Osa-miR#4 | tgagctcgggggcgaccagatc | Osa-miR#30-3p1 | aatagttctctgattacgacgcgc |
| Osa-miR#6 | aggagcagcgagaggagccaat | Osa-miR#31 | ataagacgttttgacttttt |
| Osa-miR#7 | atgtgcgcatgaaataatggaca | Osa-miR#32 | aatgtggcatattctagtact |
| Osa-miR#8 | acaggtaataaagtacgcgtgta | Osa-miR#33 | gtacactgtagcaacaatggtggc |
| Osa-miR#10 | atttgatttgtcgcgagcaat | Osa-miR#34 | atgtgacttataaattgatggcca |
| Osa-miR#11 | tccaatgcagatactgagacc | Osa-miR#35 | gaggacctgctagtttatgatggt |
| Osa-miR#12 | taaatttgatccgcacattga | Osa-miR#36 | tctgaaaactaaagaagggca |
| Osa-miR#13 | agattggtttattttgggacgg | Osa-miR#37 | tttgaaatagcgagagcgcac |
| Osa-miR#15 | taactgcatatattttggtat | Osa-miR#38 | tgaacataaagagcttagcca |
| Osa-miR#18 | ggccgttcagtaggaaccgac | Osa-miR#39 | gttacgaaacaagggagctgc |
| Osa-miR#20 | cggcgatgtgggggaggcgcg | Osa-miR#40 | tagatatttggagagtgggat |
| Osa-miR#21 | cagctgtgatgagacgtaaca | Osa-miR#42 | agaagagagagagagactgcca |
| Osa-miR#23 | actcgctggattgatcccaaa | Osa-miR#43 | tcatgtattaatgaacggagg |
| Osa-miR#24 | cgaagaggcagagagcaggatg | Osa-miR#44 | aaactttcctggtcgcgtcgg |
| Osa-miR#26 | aaaagacttcatttttagcta | Osa-miR#45 | atactttgccactttgaggag |

**Supplementary table6: Changed expression levels of miRNAs in rice ploidy lines**

**The number indicates the expression changed fold in monoploid or triploid lines compared to diplioid lines**

| **miRNA’s** | **1N/ 2N 3N/ 2N** | |
| --- | --- | --- |
| miR#3 | 1.28 | 0.84 |
| miR#8 | 1.26 | 1.64 |
| miR#9 | 1.55 | 0.92 |
| miR#12 | 1.43 | 0.98 |
| miR#17 | 2.06 | 0.96 |
| miR#23 | 1.94 | 1.03 |
| miR#25 | 1.53 | 0.90 |
| miR#28 | 1.48 | 1.05 |
| miR#29 | 1.44 | 0.71 |
| miR#30 | 1.79 | 0.89 |
| miR#38 | 1.35 | 0.79 |
| miR#43 | 1.26 | 0.55 |
| miR#45 | 1.92 | 1.09 |
